# Clinical diversity of invasive cryptococcosis in AIDS patients from central China: report of two cases with review of literature

**DOI:** 10.1186/s12879-019-4634-7

**Published:** 2019-11-27

**Authors:** Yongxi Zhang, Brian Cooper, Xi’en Gui, Renslow Sherer, Qian Cao

**Affiliations:** 1 Department of Infectious Diseases, Zhongnan Hospital of Wuhan University, 169 Donghu Road, Wuhan, 430071 China; 2 Section of Infectious Diseases and Global Health, University of Chicago, 5841 S. Maryland Avenue, MC5065, Chicago, IL 60637 USA

**Keywords:** *Cryptococcus neoformans*, *Cryptococcus laurentii*, HIV/AIDS, Opportunistic infections

## Abstract

**Background:**

Although antiretroviral therapy (ART) has greatly improved the prognosis of acquired immunodeficiency syndrome (AIDS) patients globally, opportunistic infections (OIs) are still common in Chinese AIDS patients, especially cryptococcosis.

**Case presentation:**

We described here two Chinese AIDS patients with cryptococcal infections. Case one was a fifty-year-old male. At admission, he was conscious and oriented, with papulonodular and umbilicated skin lesions, some with ulceration and central necrosis resembling molluscum contagiosum. The overall impression reminded us of talaromycosis: we therefore initiated empirical treatment with amphotericin B, even though the case history of this patient did not support such a diagnosis. On the second day of infusion, the patient complained of intermittent headache, but the brain CT revealed no abnormalities. On the third day, a lumbar puncture was performed. The cerebral spinal fluid (CSF) was turbid, with slightly increased pressure. India ink staining was positive, but the cryptococcus antigen latex agglutination test (CrAgLAT: IMMY, USA) was negative. Two days later, the blood culture showed a growth of *Cryptococcus neoformans,* and the same result came from the skin culture. We added fluconazole to the patient’s treatment, but unfortunately, he died three days later.

Case two was a sixty-four-year-old female patient with mild fever, productive cough, dyspnea upon movement, and swelling in both lower limbs. The patient was empirically put on cotrimoxazole per os and moxifloxacin by infusion. A bronchofibroscopy was conducted with a fungal culture, showing growth of *Cryptococcus laurentii* colonies. Amphotericin B was started thereafter but discontinued three days later in favor of fluconazole 400 mg/d due to worsening renal function. The patient became afebrile after 72 h of treatment with considerable improvement of other comorbidities and was finally discharged with continuing oral antifungal therapy.

**Conclusions:**

Our cases illustrate that cryptococcal disease is an important consideration when treating immunocompromised individuals such as AIDS patients. Life threatening meningitis or meningoencephalitis caused by *C. neoformans*may still common in these populations and can vary greatly in clinical presentations, especially with regard to skin lesions. Pulmonary cryptococcosis caused by *C. laurentii* is rare, but should also be considered in certain contexts. Guidelines for its earlier diagnosis, treatment and prophylaxis are needed.

## Background

Since the universal distribution of antiretroviral therapy (ART) to all human immunodeficiency virus (HIV)-infected persons regardless of CD4^+^ T-cell count, the incidence of opportunistic infections (OIs) in HIV-infected individuals appears to be in decline, especially in developed countries. But in developing countries like mainland China, OIs remain a major diagnostic sign of HIV-infection. According to a recent study, pneumocystis pneumonia and tuberculosis were still leading causes for the admission of HIV-infected patients in East China, followed by cryptococcosis. Such patients often present with very low CD4^+^T-cell counts (median = 29/μL) [1].

Members of the *Cryptococcus* genus are opportunistic encapsulated yeasts that cause significant morbidity and mortality, most often in immunocompromised hosts [2]. Clinically, without rapid and effective treatment, cryptococcal infections manifest most commonly as life threatening meningitis or meningoencephalitis. *Cryptococcus neoformans* and *Cryptococcus gattii* are the major pathogens within the genus, each with a diverse array of serotypes and genotypes [3]. Whereas *C. gattii* infections occur commonly in immunocompetent patients in endemic areas, *C. neoformans* infections remain frequent only in immunocompromised individuals [4]. Other Cryptococcus species, such as *Cryptococcus laurentii* (used as a bio-pesticide for apples), were previously believed to be saprophytic and non-pathogenic to humans, but this concept has recently been challenged [5].

Here we report two cases of cryptococcal infection in HIV/AIDS patients, one with disseminated infection preceded by generalized cutaneous lesions caused by *C. neoformans,* and the other with pulmonary cryptococcosis caused by *C. laurentii.* We then review cases of cutaneous cryptococcosis and the clinical presentations and treatments of *C. laurentii* infection from the literature.

## Case presentations

### Case 1

A fifty-year-old male was referred to our department due to newly diagnosed HIV infection, presented with a four months history of multiple skin lesions of undetermined cause affecting almost the whole body with the face involved but not the palms and soles; the lesions were pruritic, but the patient had no fever or other pathological signs. During these four months, he had received symptomatic treatment for “eczema” from other doctors, but the rashes worsened. At admission, he was conscious and oriented without headache or vomiting; other signs of meningeal irritation were also negative. Most of the skin lesions were papulonodular and umbilicated in form, some resembling molluscum contagiosum lesions with ulceration and central necrosis. He reported no other significant past illnesses. The HIV infection was due to sexual contact. He worked as a truck driver in the central and eastern regions of China and had never been to southern China, where *Talaromyces marneffei* infections are endemic.

**Fig. 1:**
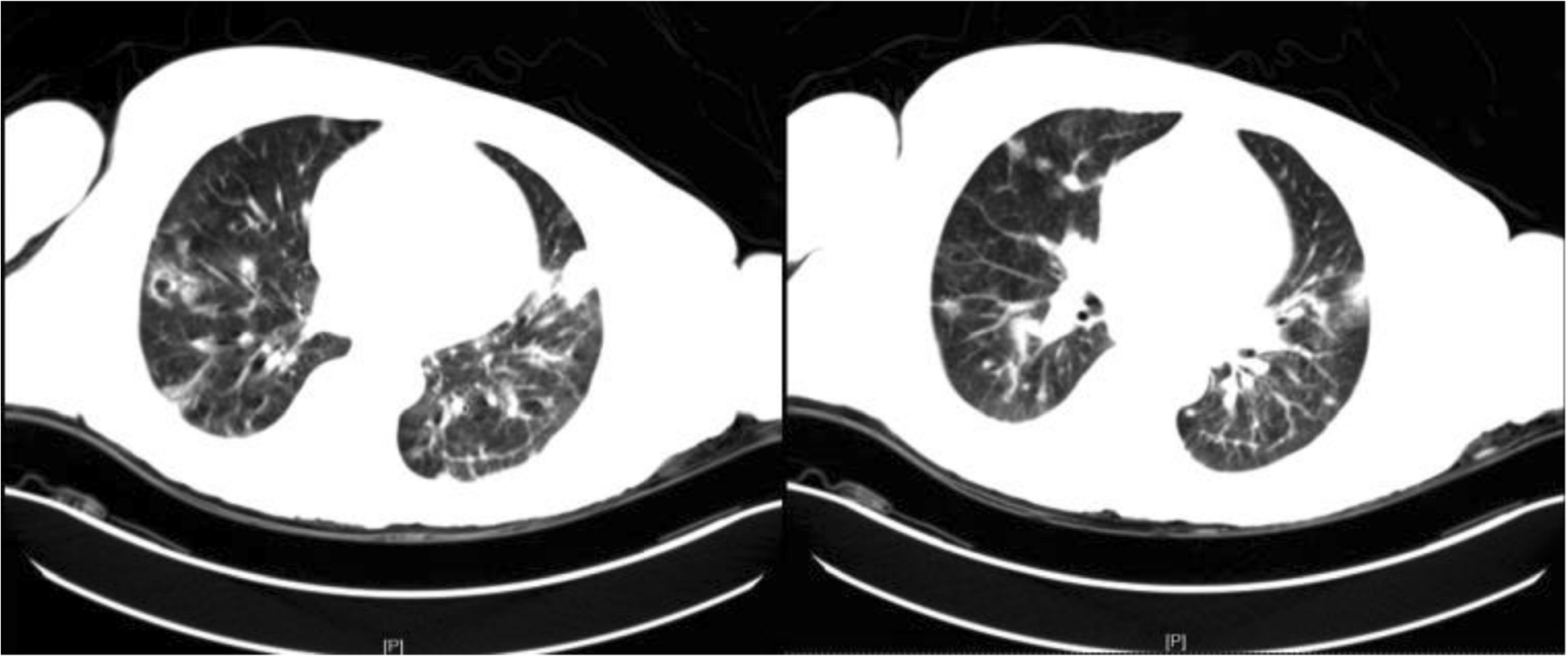
Clinical images of the patient’s skin lesions

His baseline investigation included a complete blood count, revealing a white-blood-cell count of 5.53 × 10^9^/L(*N* = 3.5–9.5) with 92.8% (*N* = 40–75) neutrophils. His hemoglobin level was 106 g/L (*N* = 130–175) and platelet count 47 × 10^9^/L (*N* = 125–350). His sodium level was 135.8 mmol/L (*N* = 137–147), whereas the potassium, chloride, bicarbonate, creatinine and urea nitrogen level of blood were within the normal value. His liver function tests were within normal limits, with albumin at 34.9 g/L (*N* = 40–55) and globulin at 43.9 g/L (*N* = 20–30). His high sensitivity C-reactive protein (hs-CRP) level was 8.26 mg/L (*N* = 0–3) and a fungal G test of plasma (Beijing Jinshanchuan Technology Development Co.,Ltd.) result was 52.27 pg/mL (*N* < 60). His CD4^+^ T-cell count was 15/μL. Chest computed tomography (CT) showed multiple mediastinal and bilateral axillary lymphadenopathy and a small amount of pleural effusion in the right chest, without obvious abnormalities in either lung. Paired blood cultures and skin-lesion biopsies plus culture were also done on the first day of admission. The morphology of the patient’s skin lesions (Fig. 1) reminded us of talaromycosis*,* which is not infrequent in our setting for we have received many patients came from southern China who has been diagnosed with *Talaromyces marneffei* and who had almost the same rashes like this patient; we therefore initiated treatment with amphotericin B (1 mg/kg), even though the epidemiological history of this patient did not support such a diagnosis. On the second day of infusion, the patient complained of intermittent headache. We suspected the headaches to be a side effect of the amphotericin B, but completed a brain CT as a precaution: it revealed no abnormalities. Upon initial presentation, we delayed performing a lumbar puncture due to concerns of an iatrogenic infection from the puncture site; however, on the third day, the patient experienced a sudden transient loss of consciousness and a lumbar puncture was performed. The cerebral spinal fluid (CSF) was turbid, with a pressure as 33 cmH_2_O. India ink staining was positive for budding round to oval encapsulated yeasts consistent with cryptococcus, but the cryptococcus antigen latex agglutination test (CrAgLAT: IMMY, USA) was negative for both cerebrospinal fluid and blood samples. Two days later, culture results confirmed the presence of *C. neoformans* (identified by both VITEK® MS IVD 3.0 and VITEK® 2 Compact System, BioMérieux) in blood and the sampled skin lesion. We added fluconazole to the patient’s treatment, but unfortunately, he died three days later.

### Case 2

A sixty-four-year-old female patient was transferred to our department due to newly diagnosed HIV infection. In the past seven months, she had been repeatedly hospitalized elsewhere for pulmonary infections and had received a variety of antibiotic treatments during her hospitalizations, including third-generation cephalosporins, fluoroquinolones, tienam and corticosteroids, all with limited effect. Her symptoms persisted, including mild fever, productive cough, dyspnea upon movement, and swelling in both lower limbs. Her HIV status was confirmed by western blot and her CD4^+^ T-cell count was 69/μL. She was a family hotel owner, reported no high-risk behaviors, and did not have any significant history of traveling, but her husband had passed away two years prior due to an unknown disease. She had been diagnosed with diabetes mellitus two years prior and was on insulin treatment, under which she had maintained a normal level of blood glucose until recently, when her glucose began showing fluctuations.

**Fig. 2:**
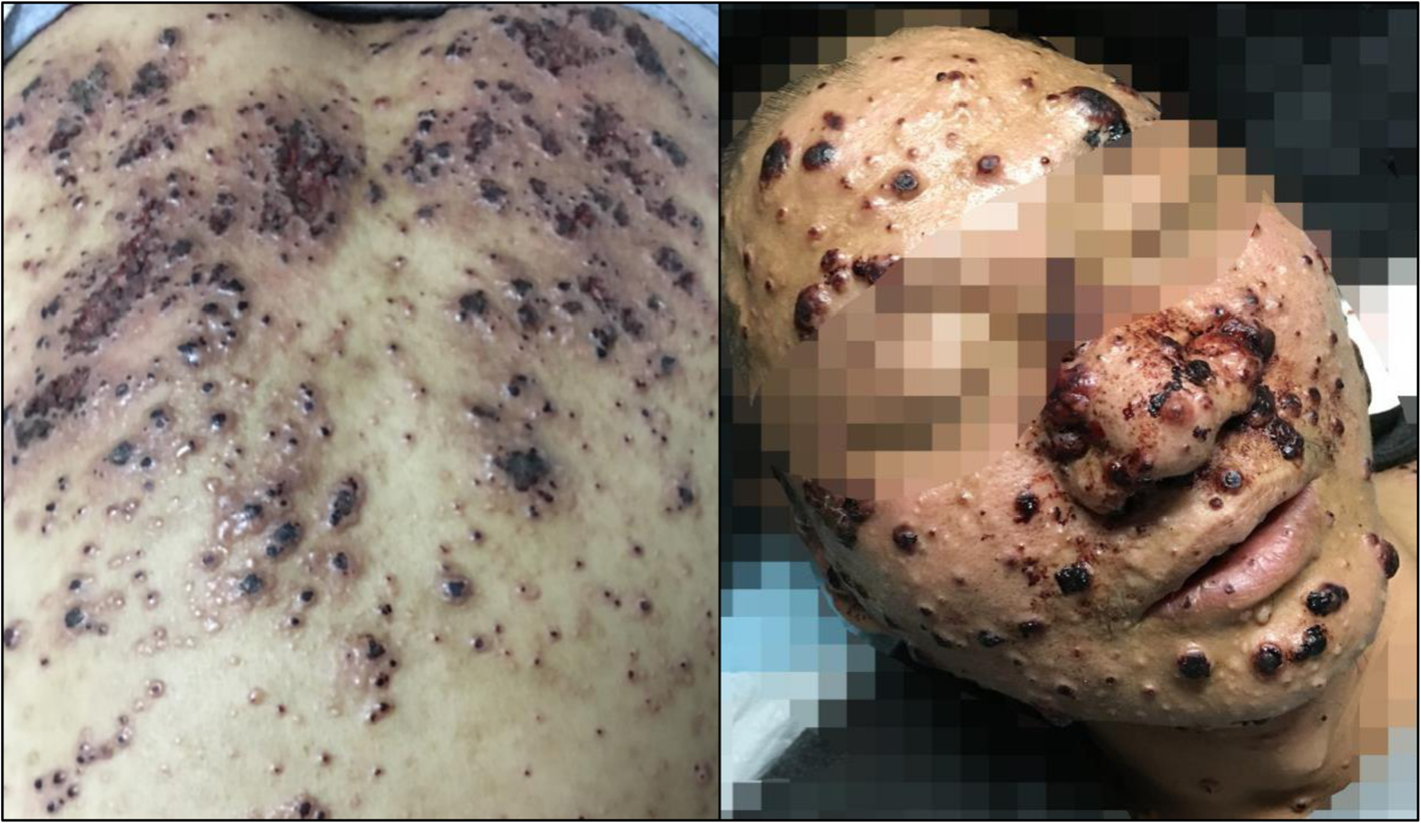
Chest CT of the patient showed features of pulmonary infection, with localized emphysema at the right lower lung, and multiple mediastinal lymphadenopathy

On examination, the patient was febrile but her vitals were normal. Auscultation demonstrated reduced breath sounds bilaterally and wet rales at the base of both lungs. The rest of the physical examination was unremarkable, except for oral leukoplakia. Laboratory investigations showed a random blood glucose of 11.2 mmol/L (*N* < 11.1), a hemoglobin level of 106 g/L (*N* = 130–175), a white-blood-cell count of 9.54 × 10^9^/L (*N* = 3.5–9.5) with 90% neutrophils (*N* = 40–75), hs-CRP at 14.7 mg/L (*N* = 0–3) and an erythrocyte sedimentation rate (ESR) of 25 mm/h (N = 0–20). Her liver function tests were within normal limits, with albumin at 20.7 g/L (N = 40–55), and her renal function was normal. Her fungal G test of plasma (Beijing Jinshanchuan Technology Development Co.,Ltd.) result was 16 pg/mL (*N* < 60). A smear for acid-fast bacillus and an interferon-gamma release assay were negative.Chest CT showed features of pulmonary infection, with localized emphysema at the right lower lung, and multiple mediastinal lymphadenopathy, suggested for the infection of *Pneumocystis jirovecii*or *staphylococcus aerus* (Fig. 2).

The patient was empirically put on cotrimoxazole per os and moxifloxacin by infusion, fluconazole 100 mg was added for the oral leukoplakia, but she still had spikes of fever. At the third day of her hospitalization, both the blood and urine culture results turned out as negative. As tuberculosis is the second most common cause of pneumonia after *Pneumocystis jirovecii* for AIDS patients in China, a therapeutic anti-tuberculosis treatment was initiated with standard quadruple regimens including isoniazid, rifampicin, pyrazinamide and ethambutol. The patient’s general condition improved, but she still had spikes of fever. We conducted a bronchofibroscopy and obtained a fungal culture that showed growth of *C. laurentii* colonies (identified by both VITEK® MS IVD 3.0 and VITEK® 2 Compact System, BioMérieux). Amphotericin B (1 mg/kg) was started thereafter but discontinued three days later in favor of fluconazole 400 mg/d due to worsening renal function (creatinine = 159.7umol/L, *N* = 49–90). The patient became afebrile after 72 h of treatment with considerable improvement of other comorbidities and was finally discharged with continuing oral antifungal therapy.

## Discussion and conclusions

The portal of entry for cryptococcus species is usually through inhalation of spores or desiccated yeast cells from the environment. In immunocompetent individuals, deposition in the alveoli produces an asymptomatic infection that is usually cleared or controlled by a strong cell-mediated immune response leading to latency. However, in immunocompromised individuals (including AIDS patients, organ transplant recipients, and those on long-term corticosteroid treatment or chemotherapy), the fungus can spread from the lungs to the central nervous system, causing meningoencephalitis, or can enter the circulatory system and disseminate hematogenously; both pathways are fatal if left untreated [6]. Cutaneous cryptococcosis is most often considered to be a clinical manifestation of disseminated disease apparent in 10–15% of patients with systemic cryptococcosis [7]. The existence of primary cutaneous cryptococcosis (PCC) has been a longstanding controversy. Neuville and colleagues defined PCC as skin lesions confined to a circumscribed body region, predominantly a solitary skin lesion on an unclothed area presenting as a whitlow or phlegmon in patients with a history of skin injury, participation in outdoor activities, or exposure to bird droppings. A positive skin culture showing *C. neoformans* with no sign of simultaneous dissemination (regional lymphadenopathy was not considered to be dissemination) is necessary to determine the infection to be primary cutaneous. Neuville et al suggest all other cases to be secondary cutaneous cryptococcosis [8]. In their nationwide (France) cohort of 1974 cases of cryptococcosis between 1985 and 2000, 108 (5.5%) had a positive skin culture, among which 28 were considered to be PCC and the other 80 to be secondary cutaneous cryptococcosis (SCC). 3 of the 28 PCC patients (11%) were HIV positive, whereas 46 of the 80 patients in the SCC group (58%) were HIV positive, suggesting PCC to be at least as prevalent in HIV-negative hosts as in HIV-positive patients, usually with a favorable prognosis among the former. According to the above definition, our patient No.1 would be classified in the SCC group, as he presented with widespread eruptions reminiscent of molluscum contagiosum at various sites without evidence of skin lesions or injuries in his recent history. Taking into account the onset of rashes in our patient, which was much earlier (four months ago) than the onset of meningeal symptoms, we speculated that in our patient, a cryptococcus fungemia had existed for a long time before presentation. The skin is an important organ affected by cryptococcosis and should be inspected for signs of dissemination. However, meningoencephalitis remains the most frequent disease presentation.

In our department, we have received a total of 64 HIV-positive patients with culture confirmed *C. neoformans* infection over the five years 2013–2017, making cryptococcosis the fifth most common OI after oral candidiasis, tuberculosis, pneumocystis pneumonia, and CMV infection, but the first cause of death (14/64, 22%) by mortality among all our AIDS patients. The median patient age was 41 years (ranging from 20 to 68) with a sex ratio of 7:1 male-to-female. The CD4^+^ T-cell count in these patients ranged from 1 to 266/μL with 92% under 100/μL and an average of 47/μL. The meningoencephalitis was the most common clinical presentation (51/64, 80%), followed by the pulmonary infection (6/64, 9%) and the blood dissemination (6/64, 9%). Only one patient (1/64, 2%) presented with cutaneous infection. Among our cryptococcosis patients, fever was the second most frequent symptom (after headache), which presented in more than half of the patients (37/64, 58%), but not in our patient in Case 1. We speculated that this might be due to the weakness of his immune system, which could not properly react against the pathogen, since his CD4^+^ T-cell count was only 15/μL. At our medical center, we strongly recommend physicians to test for HIV in all patients with suspicious uncommon skin lesions, and to consider OIs in case of HIV seropositivity.

At the same time, the Cryptococcus antigen latex agglutination test (CrAgLAT) produced a negative result in our patient with cutaneous cryptococossis (Case 1), while his culture was positive. This is a known but infrequent phenomenon that is not very well understood. Because the handling of this invasive, life-threatening pathogen is always urgent [9] and fungal cultures take time, a diagnosis is often made on the basis of a positive cryptococcal antigen (CrAg) with an appropriate clinical syndrome in the absence of a positive culture to still be definitive. The latex agglutination test or enzyme immunoassay kit is used often in our clinic. Cryptococcal antigen tests on blood and CSF are a highly reliable and rapid diagnostic technique with sensitivity and specificity > 93% [10]. False positives are generally rare but are slightly more common in HIV-positive patients due to non-specific reactions [11], or in association with rheumatoid factors in the serum, collagen vascular disease, rare infections or other malignancies [12]. False negatives have also been reported but are rare and not well characterized. Some reports have shown that they occur either with poorly encapsulated organisms and low fungal burden or due to the prozone phenomenon [13]. But other investigations have shown that the prozone phenomena or interference from bound antibodies or proteins cannot account for the false negative results [14]. Our patient’s condition deteriorated very quickly. In the last stage of his disease the fungal burden should have been very high. Unfortunately, our patient died before we could perform a sample dilution or initiate pronase treatment. Other influencing factors should be studied using different CrAg kits for different serotypes of cryptococcosis. In the case of our patient, the negative latex agglutination test did not exclude the diagnosis of cryptococcal infection: only a single specimen was tested and the patient exhibited symptoms consistent with cryptococcosis. For this reason, we strongly recommend that physicians and laboratory personnel conduct a CrAg test paired with a fungal culture using CSF and serum. In addition, the result of the CrAgLAT should be carefully interpreted with consideration of the clinical context and the possibility of false positives and negatives. The culture itself should be carefully inspected daily so that initial detection of fungal growth will not be delayed.

*C.laurentii* was formerly considered to be saprophytic and non-pathogenic to humans, but this concept has been challenged recently. Most studies have been case reports. As with our patient (Case 2), *C. laurentii* manifests clinically in a manner similar to *C. neoformans* and *C. gattii*, but the cryptococcal antigen test is often negative [15]. *C. laurentii*is prevalent worldwide and has been isolated from various environmental sources including air, soil, water, pigeon droppings and food items such as cheese, milk, beans and wine [16]. Conditions associated with impaired cell-mediated immunity are important risk factors for non-neoformans cryptococcal infections, such as old age, prior steroid exposure, prior immunosuppression, and low CD4^+^ count [17]. Other risk factors associated with *C. laurentii* infection are exposure to pigeon excrement, invasive devices and neutropenia. Prior azole prophylaxis has been associated with antifungal resistance [5].

There exists no validated standard treatment for *C. laurentii* infection. Studies correlating in vitro antifungal susceptibility and treatment outcomes do not exist. To facilitate management of our patient, we performed a literature search in PubMed using the search term “*Cryptococcus laurentii*” and identified 23 case reports in the English language from 1977 to 2017 (Table 1), 10 of which were reported over the last ten years (43.5%), although it is unclear if this is due to a true increase in the incidence of *C. laurentii* infections or an increased awareness of the pathogen among physicians and, thus, a reporting bias. Our search results showed a higher prevalence in immunocompromised hosts compared to immunocompetent hosts (22:1), with a broad geographic and age distribution (< 1 to 76 years). Populations at special risk are neonates, individuals with HIV/AIDS infection and patients being treated with corticosteroids or with prior surgeries or malignancies. The main clinical manifestations include fever (*n* = 9) with digestive symptoms (*n* = 2), respiratory symptoms (*n* = 4), signs of meningitis (n = 4), sepsis syndrome (*n* = 1), ophthalmic symptoms (n = 2) and skin lesions (*n* = 5). Amphotericin B and fluconazole alone or together were the preferable therapy for the treatment of *C. laurentii* infection. In some cases, they were used in combination with flucytosine or itraconazole. Only one patient was treated with voriconazole. Prognoses were overwhelmingly favorable: in all but one reported cases, patients recovered after as few as one week and up to ten months after beginning antifungal therapy. Our patient 2 was successfully treated with fluconazole alone, with considerable amelioration of her symptoms after initiating therapy. Only increased physician vigilance and improvements in techniques for culture and other identification methods will contribute to the early diagnosis and treatment of such unusual fungal infections. Guidelines for earlier diagnosis, treatment and prophylaxis for immunocompromised hosts are urgently needed.

**Table 1:** Clinical characteristics of cases reports of *C.laurentii* infection in English language publications on PubMed

Year	Age/gender	Region	Underlying conditions	Main manifestation	Positive culture results	Medical treatment	Duration of therapy	Outcome	Citation
2017	24/F	India	HSCT recipient	Diarrhea	Stool	Voriconazole	6 weeks	Resolved	[18]
2017	47/F	Korea	HSCT recipient	Fever +skin lesion	Blood+skin biopsy	Amphotericin B	3 weeks	Resolved	[19]
2015	30/F	India	Post-partum	Signs of meningitis	CSF	Amphotericin B	2 days	Dead	[20]
2015	42/F	Brazilian	Cervical neoplasia	Dyspnea	Blood	Fluconazole	22 weeks	Resolved	[21]
2015	74/M	Italy	Chemotherapy for rectal cancer	Nausea+vomit+diarrhea	Stool	Amphotericin B liposome	10 days	Resolved	[22]
2015	47/F	Italy	SLE	Fever	Bronchial lavage	Amphotericin B + Fluconazole	8 months	Resolved	[23]
2013	8/F	Spain	Immunocompetent	Skin lesion	Scrapings	Fluconazole	2 weeks	Resolved	[24]
2013	76/M	Indian	Post-operation	Fever +respiratory failure	Blood	Amphotericin B liposome + Fluconazole	4 weeks	Resolved	[25]
2012	55/M	Indian	Renal transplant recipient	Skin lesion	Skin biopsy	Amphoterincin B + flucytosine	> 7 days	Resolved	[26]
2009	−/M	Poland	Glomerulonephritis under immunosupressant	Fever	Blood	Fluconazole + Itraconazole	8 weeks	Resolved	[27]
2006	35/F	Indian	Diabetic +AIDS	Fever, cough, sputum	Sputum and pleural fluid	Fluconazole	5 weeks	Resolved	[28]
2006	35/M	Hawaii	AIDS	Fever +Signs of meningitis	Blood+CSF	Amphotericin B + Fluconazole	3 months	Resolved	[29]
2005	9/M	Hungary	HIGM-1	Signs of meningitis	CSF	Fluconazole	10 months	Resolved	[30]
2002	16/M	Israel	Ganglioneuroblastoma	Fever	Blood	Amphotericin B	3 weeks	Resolved	[31]
2001	< 1/F	Taiwan	Premature neonate	Sepsis syndrome	Blood	Amphotericin B	40 days	Resolved	[32]
1998	34/M	Greece	AIDS	Signs of meningitis	CSF	Amphotericin B + flucytosine	2 weeks	Resolved	[33]
1998	< 1/M	USA	Premature neonate	Urinary tract deformity	Urine + blood	Amphotericin B + flucytosine	2 weeks	Resolved	[34]
1998	27/F	USA	IDU	Fever +Skin lesion	Blood	Fluconazole	4 weeks	Resolved	[34]
1998	51/M	USA	Diabetic	Keratitis	Corneal	Amphotericin B + miconazole	14 weeks	Resolved	[35]
1997	17/M	Slovak	Bone marrow transplant	Fever	Blood	Fluconazole	2 weeks	Resolved	[36]
1995	61/F	USA	Chronic uveitis	Endophthalmitis	Vitreous biopsy	Fluconazole	5 months	Resolved	[37]
1981	–	–	Corticosteroid therapy for dermatomyositis	Lung abscess	–	Amphotericin B	–	Resolved	[38]
1977	40//M	–	MAC coinfection	Cutaneous granulomas	Skin biopsy	Amphoterincin B	–	Resolved	[39]

*HIGM-1* X-linked hyper IgM syndrome, *HSCT* allogeneic/autologous hematopoietic stem cell transplant, *IDU* intravenous drug user. *MAC Mycobacterium avium* complex, *SLE* systemic lupus erythematosus

In summary, our cases illustrate that cryptococcal disease is an important consideration when treating immunocompromised individuals such as HIV/AIDS patients. Life threatening meningitis or meningoencephalitis caused by *C. neoformans* may still common in these populations and can vary greatly in clinical presentations, especially with regard to skin lesions. Clinicians should remain alert to the possibility of fungal infections in these patients and remember that not all disease is due to *neoformans* or *gattii*, and other species should be considered when antigen testing is negative in the face of positive culture. Pulmonary cryptococcosis caused by *C. laurentii* is rare but should also be considered in certain contexts. Guidelines for its earlier diagnosis, treatment and prophylaxis are needed.

## Data Availability

Data sharing is not applicable to this article as no datasets were generated or analyzed during the current study. If reader would like additional information about the radiological images or investigations, he or she should contact the corresponding author. Any identifying information will be removed prior to sharing of images.

## Abbreviation

AIDS: Acquired immunodeficiency syndrome
ART: Antiretroviral therapy
CrAg: Cryptococcal antigen
CrAgLAT: Cryptococcus antigen latex agglutination test
CSF: Cerebral spinal fluid
CT: Computed tomography
ESR: Erythrocyte sedimentation rate
HIGM-1: X-linked hyper IgM syndrome
HIV: Human immunodeficiency virus
hs-CRP: High sensitivity C-reactive protein
HSCT: Allogeneic/autologous hematopoietic stem cell transplant
IDU: Intravenous drug user
MAC: *Mycobacterium avium* complex
OIs: Opportunistic infections
PCC: Primary cutaneous cryptococcosis
SCC: Secondary cutaneous cryptococcosis
SLE: Systemic lupus erythematosus

## References

[1] Luo B, Sun J, Cai R, Shen Y, Liu L, Wang J, Zhang R, Shen J, Lu H (2016). Spectrum of Opportunistic Infections and Risk Factors for In-Hospital Mortality of Admitted AIDS Patients in Shanghai. Medicine (Baltimore).

[2] Srichatrapimuk S, Sungkanuparph S (2016). Integrated therapy for HIV and cryptococcosis. AIDS Res Ther.

[3] Hagen F, Khayhan K, Theelen B, Kolecka A, Polacheck I, Sionov E, Falk R, Parnmen S, Lumbsch HT, Boekhout T (2015). Recognition of seven species in the Cryptococcus gattii/Cryptococcus neoformans species complex. Fungal Genet Biol.

[4] Kwon-Chung KJ, Fraser JA, Doering TL, Wang Z, Janbon G, Idnurm A, Bahn YS (2014). Cryptococcus neoformans and Cryptococcus gattii, the etiologic agents of cryptococcosis. Cold Spring HarbPerspect Med.

[5] Khawcharoenporn T, Apisarnthanarak A, Mundy LM (2007). Non-neoformans cryptococcal infections: a systematic review. Infection..

[6] May RC, Stone NR, Wiesner DL, Bicanic T, Nielsen K (2016). Cryptococcus: from environmental saprophyte to global pathogen. Nat Rev Microbiol.

[7] Christianson JC, Engber W, Andes D (2003). Primary cutaneous cryptococcosis in immunocompetent and immunocompromised hosts. Med Mycol.

[8] Neuville S, Dromer F, Morin O, Dupont B, Ronin O (2003). Lortholary O; French Cryptococcosis study group. Primary cutaneous cryptococcosis: a distinct clinical entity. Clin Infect Dis.

[9] Aye C, Henderson A, Yu H, Norton R (2016). Cryptococcosis—the impact of delay to diagnosis. ClinMicrobiol Infect.

[10] Tanner DC, Weinstein MP, Fedorciw B, Joho KL, Thorpe JJ, Reller L (1994). Comparison of commercial kits for detection of cryptococcal antigen. J ClinMicrobiol..

[11] Asawavichienjinda T, Sitthi-Amorn C, Tanyanont V (1999). Serum cyrptococcal antigen: diagnostic value in the diagnosis of AIDS-related cryptococcal meningitis. J Med Assoc Thail.

[12] Boom WH, Piper DJ, Ruoff KL, Ferraro MJ (1985). New cause for false-positive results with the cryptococcal antigen test by latex agglutination. J ClinMicrobiol..

[13] Lourens A, Jarvis JN, Meintjes G, Samuel CM (2014). Rapid diagnosis of cryptococcal meningitis by use of lateral flow assay on cerebrospinal fluid samples: influence of the high-dose "hook" effect. J ClinMicrobiol..

[14] Currie BP, Freundlich LF, Soto MA, Casadevall A (1993). False-negative cerebrospinal fluid cryptococcal latex agglutination tests for patients with culture-positive cryptococcal meningitis. J ClinMicrobiol.

[15] Hong N, Chen M, Fang W, Al-Hatmi AMS, Boekhout T, Xu J, Zhang L, Liu J, Pan W, Liao W. Cryptococcosis in HIV-negative patients with renal Dialysis: a retrospective analysis of pooled cases. Mycopathologia. 2017;30 [Epub ahead of print]. 10.1007/s11046-017-0163-3 PMC5587633 28667348

[16] Arendrup MC, Boekhout T, Akova M, Meis JF, Cornely OA, Lortholary O; European Society of Clinical Microbiology and Infectious Diseases Fungal Infection Study Group; European Confederation of Medical Mycology. ESCMID and ECMM joint clinical guidelines for the diagnosis and management of rare invasive yeast infections. ClinMicrobiol Infect. 2014 Apr;20 Suppl 3:76–98. 10.1111/1469-0691.12360 24102785

[17] Banerjee P, Haider M, Trehan V, Mishra B, Thakur A, Dogra V, Loomba P (2013). Cryptococcus laurentiifungemia. Indian J Med Microbiol.

[18] Bhat V, Vira H, Khattry N, Toshniwal M. Cryptococcus laurentii diarrhea post hematopoietic stem cell transplant. Transpl Infect Dis. 2017 Apr;19(2). 10.1111/tid.12663. 10.1111/tid.12663 28083955

[19] Park SS, Lee H, Park WS, Hwang SH, Choi SI, Choi MH, Lee SW, Ko EJ, Choi YJ, Eom HS (2017). A case of disseminated infection with skin manifestation due to non-neoformans and non-gattii Cryptococcus in a patient with refractory acute myeloid leukemia. Infect Chemother.

[20] Mittal N, Vatsa S, Minz A (2015). Fatal meningitis by Cryptococcus laurentii in a post-partum woman: a manifestation of immune reconstitution inflammatory syndrome. Indian J Med Microbiol.

[21] Neves RP, Lima Neto RG, Leite MC, Silva VK, Santos Fde A, Macêdo DP (2015). Cryptococcus laurentii fungaemia in a cervical cancer patient. Braz J Infect Dis.

[22] Calista F, Tomei F, Assalone P, Traficante D, Di Pilla G, Pepe C, Di Lullo L (2015). Cryptococcus laurentii diarrhea in a neoplastic patient. Case Rep Oncol Med.

[23] Conti F, Spinelli FR, Colafrancesco S, Truglia S, Ceccarelli F, Fattapposta F, Sorice M, Capozzi A, Ferretti G, Priori R, Martinelli F, Pirone C, Alessandri C, Valesini G (2015). Acute longitudinal myelitis following Cryptococcus laurentii pneumonia in a patient with systemic lupus erythematosus. Lupus..

[24] Molina-Leyva A, Ruiz-Carrascosa JC, Leyva-Garcia A, Husein-Elahmed H (2013). Cutaneous Cryptococcus laurentii infection in an immunocompetent child. Int J Infect Dis.

[25] Banerjee P, Haider M, Trehan V, Mishra B, Thakur A, Dogra V, Loomba P (2013). Cryptococcus laurentii fungemia. Indian J Med Microbiol.

[26] Kulkarni A, Sinha M, Anandh U (2012). Primary cutaneous cryptococcosis due to Cryptococcouslaurentii in a renal transplant recipient. Saudi J Kidney Dis Transpl.

[27] Furman-Kuklińska K, Naumnik B, Myśliwiec M (2009). Fungaemia due to Cryptococcus laurentii as a complication of immunosuppressive therapy--a case report. Adv Med Sci.

[28] Shankar EM, Kumarasamy N, Bella D, Renuka S, Kownhar H, Suniti S, Rajan R, Rao UA (2006). Pneumonia and pleural effusion due to Cryptococcus laurentii in a clinically proven case of AIDS. Can Respir J.

[29] Khawcharoenporn T, Apisarnthanarak A, Kiratisin P, Mundy LM, Bailey TC (2006). Evaluation of cryptococcuslaurentii meningitis in a patient with HIV infection: a case report and review of the literature. Hawaii Med J.

[30] Simon G, Simon G, Erdös M, Maródi L (2005). Invasive Cryptococcus laurentii disease in a nine-year-old boy with X-linked hyper-immunoglobulin M syndrome. Pediatr Infect Dis J.

[31] Averbuch D, Boekhoutt T, Falk R, Engelhard D, Shapiro M, Block C, Polacheck I (2002). Fungemia in a cancer patient caused by fluconazole-resistant Cryptococcus laurentii. Med Mycol.

[32] Cheng MF, Chiou CC, Liu YC, Wang HZ, Hsieh KS (2001). Cryptococcus laurentii fungemia in a premature neonate. J ClinMicrobiol..

[33] Kordossis T, Avlami A, Velegraki A, Stefanou I, Georgakopoulos G, Papalambrou C, Legakis NJ (1998). First report of Cryptococcus laurentii meningitis and a fatal case of Cryptococcus albiduscryptococcaemia in AIDS patients. Med Mycol.

[34] Johnson LB, Bradley SF, Kauffman CA (1998). Fungaemia due to Cryptococcus laurentii and a review of non-neoformans cryptococcaemia. Mycoses..

[35] Ritterband DC, Seedor JA, Shah MK, Waheed S, Schorr I (1998). A unique case of Cryptococcus laurentii keratitis spread by a rigid gas permeable contact lens in a patient with onychomycosis. Cornea..

[36] Krcméry V, Kunova A, Mardiak J (1997). Nosocomial Cryptococcus laurentii fungemia in a bone marrow transplant patient after prophylaxis with ketoconazole successfully treated with oral fluconazole. Infection..

[37] Custis PH, Haller JA, de Juan E Jr. (1995). An unusual case of cryptococcal endophthalmitis. Retina..

[38] Lynch JP, Schaberg DR, Kissner DG, Kauffman CA (1981). Cryptococcus laurentii lung abscess. Am Rev Respir Dis.

[39] Kamalam A, Yesudian P, Thambiah AS (1977). Cutaneous infection by Cryptococcus laurentii. Br J Dermatol.

