# Correction to: Clinical diversity of invasive cryptococcosis in AIDS patients from central China: report of two cases with review of literature

**DOI:** 10.1186/s12879-019-4698-4

**Published:** 2019-12-19

**Authors:** Yongxi Zhang, Brian Cooper, Xi’en Gui, Renslow Sherer, Qian Cao

**Affiliations:** 1 Department of Infectious Diseases, Zhongnan Hospital of Wuhan University, 169 Donghu Road, Wuhan, 430071 China; 2 Section of Infectious Diseases and Global Health, University of Chicago, 5841 S. Maryland Avenue, MC5065, Chicago, IL 60637 USA

**Correction to: BMC Infect Dis**

**https://doi.org/10.1186/s12879-019-4634-7**

After publication of the original article [1], we were notified that Figs. 1 and 2 has been misplaced. Hence, the position of the two pictures should be reversed.

Below you can find the figures in the correct sequence.

**Fig. 1:**
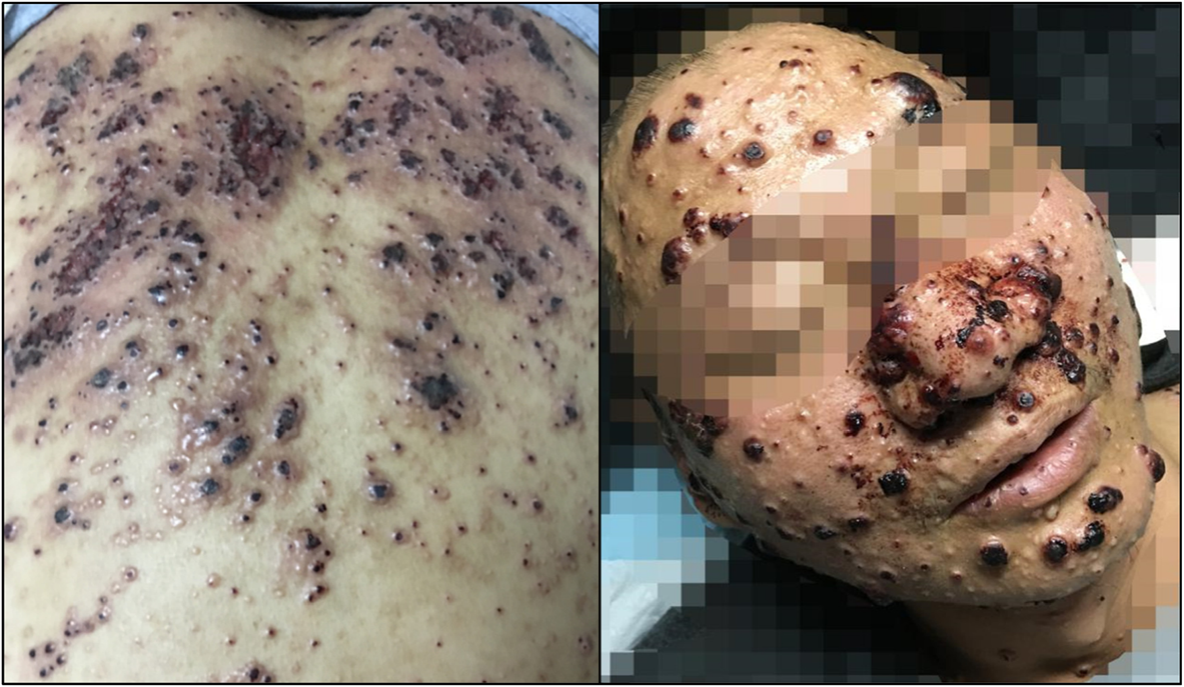
Clinical images of the patient’s skin lesions

**Fig. 2:**
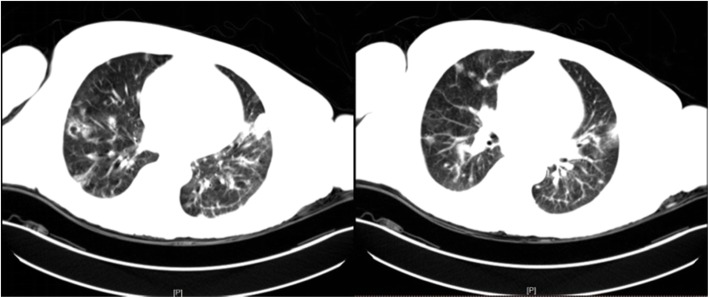
Chest CT of the patient showed features of pulmonary infection, with localized emphysema at the right lower lung, and multiple mediastinal lymphadenopathy

The original article has been corrected.

## References

[1] Zhang Y (2019). Clinical diversity of invasive cryptococcosis in AIDS patients from central China: report of two cases with review of literature. BMC Infect Dis.

